# Microwave-Energy-Based Device for the Treatment of Cellulite and Localized Adiposity: Recommendations of the “Onda Coolwaves” International Advisory Board

**DOI:** 10.3390/bioengineering11121249

**Published:** 2024-12-09

**Authors:** Klaus Hoffmann, Elena Zappia, Paolo Bonan, Federica Coli, Luigi Bennardo, Matteo Tretti Clementoni, Valerio Pedrelli, Domenico Piccolo, Irina Poleva, Benedetta Salsi, Cristina Cuciti, Maria Stella Tarico, Cinzia Incandela, Carmine Andrea Nunziata, Francesco D’Andrea, Elisabetta Perosino, Simona Carpagnano, Paola Abramo, Angeline Annine Yong, Renato Soriani Paschoal, Maria Daniela Villavicencio Romero, Aura Ibeth Ruiz Rosas, Daniel Ricardo Galimberti, Athina Matekovits, Susanne Hjøllund Kjeldsen, Tina Jensen, Paolo Mezzana, Heba Msallam, Simone Ribero, Steven Paul Nisticò

**Affiliations:** 1 Department of Dermatology, Ruhr-University, 44787 Bochum, Germany; klaus.hoffmann@klinikum-bochum.de; 2 Department of Health Sciences, Magna Graecia University, 88100 Catanzaro, Italy; 3 Department of Medical Sciences, University of Turin, 10126 Turin, Italy; 4 Laser Cutaneous Cosmetic & Plastic Surgery Unit, Villa Donatello Clinic, 50121 Florence, Italy; 5 Laserplast Clinic, 20100 Milan, Italy; 6 Skin Centers, 65121 Pescara, Italy; 7 CGH Clinic, 00100 Rome, Italy; 8 Division of Dermatology, Poliambulatorio San Michele, 42121 Reggio Emilia, Italy; 9 Unit of Dermatology, San Donato Hospital, 52100 Arezzo, Italy; 10 Complex Operating Unit of Plastic Surgery, A.O. Cannizzaro, 95126 Catania, Italy; 11 Private Practice, 90100 Palermo, Italy; 12 Clinica Estetica Cimarosa, 80100 Naples, Italy; 13 Department of Plastic Surgery, University of Naples, 80100 Naples, Italy; 14 Poliambulatorio Medicalia, Via Sofia 34, 00100 Rome, Italy; 15 Private Practice, 76121 Barletta, Italy; 16 Private Practice, 88100 Catanzaro, Italy; 17 Gleneagles Medical Centre, Singapore 258500, Singapore; 18 RS Academy, Private Institution, Ribeirao Preto 14000-000, Sao Paulo, Brazil; 19 Universidad de Cuenca, Cuenca 010201, Ecuador; 20 Department of Dermatology, National University of Colombia, Bogotá 111321, Colombia; 21 Derma International, Buenos Aires 2456, Argentina; 22 School of Medicine, National and Kapodistrian University of Athens, 157 27 Athens, Greece; 23 Klinik 81, 7400 Herning, Denmark; 24 Skin Differenz, 1850 Frederiksberg, Denmark; 25 Delle Medical Center, 00100 Rome, Italy; 26 Private Practice, Gaza P840, Palestine; 27 Department of Dermatology, Sapienza University, 00100 Rome, Italy

**Keywords:** microwave technology, skin laxity, cellulite reduction, body contour

## Abstract

The body contour market has grown rapidly in recent years, due to persistent requests for noninvasive treatments for localized fat adiposities, cellulite, and skin laxity. A variety of different methods are now available to improve body shaping. This review aims to provide an exhaustive compendium of the main recommendations for the optimal use of an innovative device delivering microwaves (MWs) for unwanted fat and cellulite reduction (Onda Coolwaves, DEKA, Florence, Italy), resulting from the experiences of the most expert international users. The availability of this new technology has led to an increasing number of treated patients and clinical studies. However, what is still missing, to the best of our knowledge, is an evaluation of the long-term efficacy and safety of this method. Based on the most recent data available, this compendium focuses on the ideal parameters, patient selection, and treatment methodology for providing safe and effective treatment protocols. Future research findings may suggest changes to the conclusions or recommendations in this report.

## 1. Introduction

Microwave technology is very popular nowadays, but it is not a novelty in medical applications. New devices have been introduced in the aesthetic medicine market. Among these, an innovative device delivering microwaves (MWs) (Onda Coolwaves, DEKA, Florence, Italy) has demonstrated efficacy and safety for use in aesthetic dermatological indications, such as body contouring and the treatment of cellulite and skin laxity [1].

Cellulite is a common and aesthetically distressing skin condition characterized by dimples and depressions that create an uneven skin surface. Affecting 80% to 90% of women, primarily on the thighs, buttocks, and hips, cellulite is associated with significant negative psychosocial effects and a reduced quality of life. The etiopathogenesis of cellulite is multifactorial and complex, involving alterations in subcutaneous fat and connective tissue, which are not yet fully understood [2]. The device under review operates at a frequency of 2.45 GHz, delivering power levels up to 200 W. It utilizes two specialized handpieces: a deep applicator and a shallow applicator. The deep handpiece is designed to target and heat deeper subcutaneous tissues, while the shallow handpiece focuses on more superficial layers, enhancing skin tightening and remodeling effects. An integrated cooling system is incorporated into both handpieces to protect the epidermis, preventing excessive heating of the skin’s surface and ensuring patient comfort during the procedure.

The appropriate selection of microwave frequency and technology can determine the correct depth, so that subcutaneous fat cells can be targeted effectively and safely [3]. A microwave frequency of 2.45 GHz, in combination with the special shape of the coaxial emission handpiece, can be directed over the subdermal fat layer specifically for aesthetic medicine treatments. Moreover, contact skin cooling protects the superficial layers of the dermis from overheating.

Microwaves subject adipocytes to significant stress, causing metabolic alterations. These changes prompt the adipocytes to release some of their lipid content into the cellular interstitium through a process known as “blebbing”; the fat droplets reach the plasma membrane, where they are enveloped by protrusions of the membrane, producing a bubble-like appearance in the cell. Once detached from the adipocyte, these bubbles carry the lipid content into the surrounding interstitial connective tissue. This mechanism is so intense that it induces a lysis process in the fat cells, leading to the rupture of the adipocyte membrane. The large quantity of fat droplets that spill into the interstitial connective tissue attracts macrophages from the bloodstream, which act to “cleanse” the cellular interstitium of excess fatty acids. Once normal conditions are restored, the macrophages migrate into the lymphatic system.

Clinical indications for the use microwaves include localized adiposity, cellulite, and skin laxity [1].

Cellulite is a multifactorial condition that benefits from a combination treatment approach. Histopathologically, three evolutionary stages can be identified [4]:

(1) Changes in adipocytes with lymphatic congestion and fibrocyte proliferation.

(2) Fibroplasia, collagen formation, and new capillary growth, accompanied by localized microhemorrhages and follicular hyperkeratosis. There is mild edema in the dermis. Large fibrous shoots of collagen lead to a constriction of the natural lobulation of adipose tissue, as well as adipocyte hypertrophy and water retention. This causes a typical “orange peel” appearance.

(3) The third stage includes the previously mentioned changes, along with the hardening of the fibrous septa in the subcutaneous tissue and deep dermis, resulting in a padded appearance. A granular feel, upon deep palpation of the affected area, corresponds to the nodules in the subcutaneous tissue that are observed histopathologically.

Microwaves work on cellulite by acting on multiple factors: increasing blood circulation, realigning collagen fibrils, thickening collagen fibers in the dermis, releasing messenger enzymes from adipocytes, acting on the adipocytes’ membranes (lipolysis), and rupturing and solubilizing collagen fibers. This results in the removal of the tight, nonelastic mesh constricting the lobules. Finally, the microwaves stimulate neo-collagenesis (the solubilization of collagen). In addition to reducing the “pitted appearance” of the skin, the latter may also reactivate fibroblasts, which are stimulated to generate new, increasingly elastic collagen.

Ultimately, heat produced by microwaves in the dermis may induce immediate collagen shrinkage with consequential skin tightening. The final effect is the improvement of skin laxity.

Various studies have been carried out with the Onda Coolwaves system and have proven its effectiveness and safety. The initial pilot study carried out on a group of 12 volunteers with localized fat deposits, indicated the potentially promising role of microwaves in treating localized subcutaneous fat accumulations [1]. During the microwave treatments, patients with abdominal adiposities saw a progressive reduction in their abdominal circumference. Four weeks following the final session, a median reduction of 3.90 cm (range: 7–1.5 cm) was observed. Four weeks after the treatment, volunteers with fat deposits in the trochanteric region experienced a median reduction of 2.8 cm (range: 2.5–3 cm). There were no significant differences in BMI among the volunteers. Upon evaluating the blood tests, none of the values showed significant changes. On a scale from 1 to 5, the patients agreed (4.1) that the treatment results were satisfactory. There were no reported adverse events.

A further study [3], conducted on 49 patients, evaluated the effects of microwave technology on the abdominal obesity and anthropometric indices of overweight adults, concluding that this procedure had a significant reductive effect on the crude values of the anthropometric indices. Therefore, it can be considered a safe and effective method for improving skin appearance and reducing subcutaneous fat.

A recent study [5] evaluated the safety and efficacy of microwaves for the treatment of submental skin laxity and fat. A total of 48 adult subjects underwent six treatment sessions, spaced two weeks apart. Submental treatment was performed using the shallow handpiece. A follow-up was conducted six months after the final treatment. All the examined parameters for evaluating the submental fat, the submental skin laxity, and pain had improved, confirming the Onda system’s efficacy and its safety for the submental area.

Zerbinati et al. [6] discovered new morphological evidence of structural changes in compact collagen forming interlobular septa in subcutaneous adipose tissue, in an animal model. Cellulite is structurally determined by highly compact fibrotic septa that divide large clusters of adipose tissue into tethered compartments, resulting in the typical dimpling/orange peel pattern. Morphological and morphometric data obtained after Onda treatment, using Picrosirius red staining and circularly polarized microscopy, provide supporting evidence showing that activation of a remodeling mechanism of the interlobular septa occurs with a consequent reduction in compacted fibrous collagen bundles. This study concluded that the compaction of collagen fibers, constituting fibrotic septa fragmentation and remodeling, began a few hours after microwave treatment.

Bonan et al. [7] enrolled a total of 15 women to evaluate the efficacy and safety of microwaves for treating edematous fibrosclerotic panniculopathy and skin laxity of the buttocks and/or thighs. The patients received four treatment sessions, one per month. Anthropometric measurements, BMI, cellulite severity scale (CSS) total score, morphology/number/site and depth of depressions, grade of skin laxity, and severity of EEP were collected, as well as clinical and digital photographs and 3D imaging with quantitative volume measurements. To assess comfort and satisfaction, questionnaires using a five-point Likert Scale were administered during an eight-week follow-up (t3). At t3, there was a significant improvement in both EEP severity and skin laxity. There was no mention of or assessment of pain or side effects. At t3, an average 3.3 score was assigned based on the patient satisfaction scale, indicating overall satisfaction.

These preliminary findings indicate that Onda Coolwaves is a safe, effective, and well-tolerated treatment for EEP and skin laxity, even in patients who are globally sedentary or smokers (90% and 40%, respectively).

A total of 26 women with severe or moderate cellulite underwent four sessions of microwave therapy on their buttocks and thighs [8]. Both the Cellulite Severity Scale (CSS) and the Nürnberger–Müller classification scale showed that the treatment positively impacted cellulite severity. The CSS indicated a significant improvement in cellulite severity for the buttocks and posterior thighs between the initial evaluation and the three-month follow-up, along with a notable enhancement in patient satisfaction and a reduction in the circumference of the buttocks and posterior thighs. According to the Nürnberger–Müller classification, at the three-month follow-up after the final treatment, there was a significant increase in the percentage of women with grade I/0 cellulite and a considerable reduction in the percentage of patients with grade II/III cellulite.

Two studies have assessed the effects of treatment with Onda, focusing on both efficacy and safety. These studies included follow-up periods at nine months [9] and one year [10], respectively, and reported no long-term side effects within the indicated timeframes.

Nisticò et al. [11] evaluated the efficacy of combined microwaves and flat magnetic stimulation to treat abdominal localized adiposity and laxity, carrying out a synergic action on connective and muscle tissue. This was based on their experience with the efficacy of microwaves on subcutaneous fat and skin laxity, and involved a new electromagnetic stimulation technology, flat magnetic stimulation (FMS). A total of 25 patients were subjected to two microwave treatment sessions per month on the abdominal area. FMS (Schwarzy^®^ system, DEKA, Florence, Italy) was also performed twice per week for two months, with a minimum of two days between sessions. The results show that combining microwaves and FMS treatment is safe and effective for treating abdominal subcutaneous fat and skin laxity. The treatment, which acts on both muscle cells and adipocytes, has the potential to improve body shape without being invasive.

## 2. Recommendations for the Use of the “Onda Coolwaves” Microwave Technology

### Patient Selection

First and foremost, physicians should carry out a correct and detailed examination and medical history of the patient to assess their suitability for treatment and, in each case, the most appropriate protocol.

The primary data to consider when formulating the most appropriate treatment plan include the patient’s anamnesis, a correct examination, and an analysis of the patient’s motivations and expectations.

The patient should be fully briefed on the treatment process, the results that they can expect according to their individual characteristics, the approximate number of sessions required to attain the desired results, guidelines to adhere to before and after each treatment session, and any potential side effects. Possible side effects and complications should be discussed in detail and filled out on the consent form before the treatment starts.

Anamnesis is essential to uncover any illnesses, risk factors, and potential triggers in the patient’s history that may contribute to the development of localized adiposity issues, cellulite, fluid retention, obesity, etc. (for instance: hormonal imbalances, metabolic changes, lifestyle, consumption and overconsumption of alcohol, smoking, etc.). This is very important for accurately determining the treatment inclusion criteria or, alternatively, any potential contraindications for the treatment.

The main data collected should include those described in Table 1.

A key factor in the treatment outcome is a thorough assessment of the subject’s overall physical health. Additionally, it is important to perform a detailed objective examination of the entire area to be treated, and to provide either a qualitative or quantitative evaluation of the tissue (Table 2).

Palpation is also suggested and, more specifically, the following examinations: pinching the tissue with fingers and attempting a skin detachment to assess tissue fibrosis and/or flaccidity; verifying the presence or absence of pain upon palpation (essential for detecting the degree of cellulite); evaluating the skin temperature (very hot = inflammation, very cold = circulatory slowdown); and palpating the area to detect the possible presence of oedema, muscle tone, and any abnormalities such as nodules.

During the thorough physical examination, surgical scars in the treatment area, abdominal herniation, contour deformity, and skin laxity grade should all be noted. After microwave treatment, previously unrevealed surgical scars (such as those from a caesarean section or appendectomy) may function as ledges, significantly altering the overall body contour. Likewise, severe skin laxity will not be enhanced and will persist as a deformity, and excessive removal of localized fat may lead to lax skin that was not evident prior to the volume reduction.

During the patient’s interview and examination, it is essential to gather detailed information not only concerning symptoms but also about motivations and treatment objectives.

Patient outcomes and satisfaction depend on proper patient selection. Many patients are becoming less tolerant of the risks and lengthy recovery times associated with surgical options, and they prefer treatment that does not interfere with their daily activities. If it is convenient for them, these patients are also happy with more modest improvements. Thus, the most important factor in determining patient satisfaction, especially with noninvasive devices, is realistic patient expectations. Patients should always be evaluated for unrealistic expectations. Dissatisfied patients may not have had realistic expectations about the degree of improvement that noninvasive procedures could provide. If they expected all the fat/cellulite/laxity to disappear, this would explain their dissatisfaction.

Patients with realistic expectations are the best candidates for these treatments. If the patient expects a natural, moderate improvement, they will be satisfied.

Patients who are surgery-averse, poor surgical candidates, young patients with minimal laxity, or patients who have previously had surgery and are redeveloping localized adiposity or laxity, are all excellent candidates. Patients who require additional tightening after liposculpture are also exceptional candidates.

In general, the ideal patient for microwave treatment should not be obese. If the patient is overweight, it is important to consider the distribution of fat in their body (often, in patients with a high BMI, most of their fat accumulates in a particular part of the body, whether upper or lower; for this reason, the possibility of treating a patient that is reported to be obese, according to their BMI measurement, with microwaves should not be dismissed immediately, and the area to be treated should be evaluated). This patient may have adiposity in small areas such as the abdomen, hips, buttocks, and thighs. They can present cellulite problems at different stages (in general, better results are obtained in patients with second-degree or third-degree cellulite). As for skin laxity, patients who can be treated with microwaves should not have muscle involvement. The most appropriate candidates for skin tightening of the body are those who have mild or mild to moderate skin laxity without underlying structural ptosis.

Younger patients may respond better than older patients; however, older patients with good overall skin quality may respond just as well as their younger counterparts.

Furthermore, premenopausal women and those receiving estrogen supplementation exhibit the most significant improvement in laxity after microwave treatments. This improvement may be attributed to estrogen’s direct enhancement of fibroblast activity and its role in promoting collagen and elastin production [12].

All information acquired during the patient examination should be carefully evaluated. Once analyzed, the suitability for treatment with microwaves will be determined, and the most appropriate protocol will be defined.

## 3. Exclusion Criteria

As mentioned above, after the initial consultation with the patient, the physician should determine whether the treatment is suitable, or if it should be postponed or completely ruled out.

The following paragraph indicates the main contraindications that we recommend taking into consideration when evaluating patients for treatment with the Onda system. These are general recommendations. The final decision regarding whether to carry out the treatment is, in any case, left to the medical operator based on the unique characteristics of each specific patient.

The listed “relative contraindications” are conditions and/or diseases that, based on their specific anatomic position, severity, and characteristics, may constitute a reason for the physician to postpone the treatment with microwaves or exclude it.

For patients currently on pharmacological therapy, it is crucial to investigate possible contraindications and side effects. This evaluation may lead to the decision to appropriately discontinue the therapy before beginning treatment (Table 3). Furthermore, certain areas should be excluded from treatment (Table 4). Areas can be excluded for two primary reasons: anatomical location restrictions and skin pathologies in the area to be treated.

Special attention is required when tattoos or permanent make-up are present in the treated area. A tattoo may be considered a full-fledged skin wound. Infections or allergic reactions may be caused by pigment components introduced into the skin. It is therefore strongly recommended not to treat the tattooed area until it is completely healed (which usually occurs within 20–30 days of execution).

After 4–6 months, pigments progressively move from the more superficial layers of the skin to the dermis. Furthermore, in this phase, treatment on tattooed skin is not advised. In fact, a tattoo may be considered a scar with colored pigment, often composed of unknown ingredients. External stresses such as heat or rubbing can sensitize and irritate the tattooed part. Although rare, a healed tattoo may be prone to swelling or itching for months or years after its execution, especially in periods of greater fatigue, or when the general state of health is more debilitated. For this reason, it is suggested to always examine a tattoo very carefully before evaluating whether it is appropriate to pass over it with a handpiece and, in any case, to constantly monitor reaction to the treatment.

Furthermore, if any presence of nodules, lipomas, pre-existing fibrotic tissue or any anomaly is recorded during preliminary palpation screening, we recommend proceeding with detailed diagnostic tests (ultrasound examination or other instrumental examinations, depending on the case) before treatment. This should be performed in order to identify and rule out any injuries or illnesses that might be included on the list of contraindications for the treatment.

If pain is detected during preliminary palpation screening (often associated with more advanced stages of cellulitis), it is important to both accurately identify and evaluate the cause.

It is also advisable to carry out blood tests to correctly identify the treatment inclusion criteria and to better identify any possible contraindications.

## 4. Pre-Treatment Recommendations

Before treatment, it is important to draw topographic contour markings using a permanent marker and to take photographs for documentation (see the following paragraph for more details). Marking areas to avoid or areas that require extra caution should be carried out with care (subcostal margin, iliac crest, gluteal crease). Some providers will also mark areas where more extensive fat reduction may be desired, such as the rectus abdominis border. It is essential to notice asymmetry. Scars and previous surgical sites must be clearly marked.

Due to the high absorption rate of microwaves by water, it is advised to discontinue the use of moisturizing and softening creams and gels on the area to be treated approximately one day before the session with Onda. This prevents excessive absorption of Onda Coolwaves into the skin’s superficial layers, and enhances penetration into the adipose tissue. Additionally, the patient should consume 2 L of water daily to aid in the drainage of interstitial fluids. Appropriate diet and moderate physical exercise are also suggested.

Before starting the treatment, a correct cleansing of the area to be treated and the removal of any impurities that might interact with or constitute an obstacle to treatment are advised. Removal of makeup, lotions, deodorants, or ointments with a mild soap and shaving improves the coupling between the handpiece and the skin.

The areas should be divided into subareas of 15 × 15 cm.

In the case of localized fat, it is recommended to measure the skinfold within square subareas while the patient is in a standing position.

The patient should assume the correct position on the treatment bed. To increase the adipose tissue thickness, the patient should be positioned with their torso slightly bent forward (about 45°).

It is recommended to apply a thin layer of pure Vaseline oil over the entire treatment area to ensure proper contact of the handpiece with the skin and to facilitate smooth movements. Repeat the application of the product if necessary.

The patient should not wear acrylic fiber garments during the treatment. The removal of any hearing aids may be recommended. The same area should not be treated more than once in the same session, and more than six square areas of 15 × 15 cm^2^ should not be treated in the same session (in order not to overload the lymphatic system).

Acquiring photographic images and measuring the circumferences to document the patient information in the various treatment phases helps to monitor the effectiveness of the treatment. For better photographic and measurement quality, standardize the method of taking pictures, so that the patient position, lighting conditions, and biometric reference points can be reproduced.

## 5. Methods: Ideal Setting of Parameters According to the Patients’ Needs

First and foremost, these international recommendations offer guidelines and suggestions on how to utilize the system, yet they cannot be a substitute for the clinical judgment of the physician conducting the treatment. The information provided should be regarded as a recommended usage of the device. It is not intended to serve as a complete and exhaustive guide for operating the device, and cannot replace the experience and observational skills of the operator.

### 5.1. Handpiece Selection

The Onda Coolwaves system, a novel microwave technology, is equipped with two standard handpieces deliberately designed with a coaxial emitter to limit any possible electromagnetic field dispersion. The emitter is designed to interact with proximal layers and not radiate into space like an antenna or microwave oven. This allows effective depths to be reached in a completely safe way.

Moreover, if the input impedance of the tissue does not match the emitter, the applied power is reflected from the antenna and, hence, not deposited in the tissue [13]. This can be used to produce an emission of microwaves on the fatty tissue only when a perfect coupling with the skin is achieved. If used incorrectly, an emission can be generated, increasing the safety of the system.

Additionally, the continuous cooling feature integrated into the contact handpieces helps to protect the more superficial layers of the skin from unwanted overheating.

In greater detail, the shallow handpiece generates a more concentrated and superficial heat that produces controlled hyperthermia, aimed at solubilizing fibrous collagen and inducing the contraction of the most superficial collagen fibers. This is carried out to achieve both a tightening and remodeling effect on the superficial connective tissue. Conversely, the deep handpiece produces broader and deeper heating that results in controlled hyperthermia. This effect causes molecular oscillation in the adipocytes and solubilizes the deeper collagen fibers, leading to the lipolysis of fat cells and the remodeling of collagen fibers through the activation of fibroblasts (Table 5).

### 5.2. Suggested Treatment Protocol

Based on the evaluation of the patient, the physician may choose the most suitable protocol to treat the imperfections found. On average, the complete treatment protocol consists of four sessions of about 30–60 min per area of approximately 500 cm^2^ (two squares of 15 × 15 cm^2^), to be carried out with a frequency of one session every 4 weeks.

The suggested setting of parameters according to the different clinical indications is reported here. Ranges of values are shown for both the power and dose to be selected. It is strongly recommended to initially select the minimum values of these ranges, especially for beginner users. Only later, by gaining experience and always observing all the recommendations provided in this document, will it be possible to progressively increase the levels of power and/or dose when the situation requires it.

Specifically, cellulite may benefit from a power of 130–180 W and an energy dose of 140,000–170,000 J. The lowest values of power and dose are recommended in cases of mild and quantitatively poor first-stage cellulite; on the contrary, maximum values are proposed for the most serious cases, such as third/fourth-stage fibrous cellulite.

In the case of localized adiposity, we recommend a power of 120–160 W and a dose of 120,000–150,000 J.

For small adiposities, the lowest parameters are recommended, while for more significant volumes, the dose and power may be increased. In case of skin laxity, a power of 120–150 W and a dose of 110,000–150,000 J are suggested.

If the area to be treated is “empty”, with very little adiposity, lower parameters are recommended; otherwise, if the marked laxity is accompanied by adipose panniculus, higher parameters are preferred.

Increasing the power level may increase the risk of side effects. For this reason, before selecting the maximum values indicated for power, it is recommended to initially increase only the dose (i.e., the treatment time).

In case of mixed conditions, the operator should evaluate the predominant imperfections of the area to be treated, and select the most suitable protocol starting from there.

Different indications are recommended for the submental treatment: power between 60 and 90 W, and dose between 50,000 and 70,000 J.

The submental treatment should be performed using the shallow handpiece. The only pre-platysma fat that can be treated is that in the submental area, starting from 1.5 cm below the lower border of the mandible up to the hyoid bone. This is to avoid treating the area of the marginal mandibular nerve, arteries, and veins.

Table 6 summarizes the indications for which each handpiece is recommended to be used, for the treatment of patients according to their conditions.

### 5.3. Procedure

The correct patient positioning during the treatment is of fundamental importance.

We suggest positioning the patient so that the tissue to be treated is moved as far away as possible from the muscles and bones. For example, for abdomen treatments, it is advisable to position the patient with their torso slightly bent forward. This positioning helps to increase the thickness of the adipose tissue and distance it from the muscle surface, optimizing the treatment’s effectiveness.

General indications:

- Abdomen: back slightly raised on the couch.
- Inner thigh: keep the leg to be treated straight and extended, while the other leg bends “like a butterfly” to create adequate space to move the handpiece.
- Hips and bra line: patient prone, with arms extended along the body.
- Arms: patient prone, with arms along the body and elbows flexed at approximately 90 degrees.

#### 5.3.1. Treatment Procedure

The treatment procedure may benefit from the following recommendations: skin cooling temperature of 5 °C; treatment of a square area (15 × 15 cm^2^); and circular/wavy and linear movements (like a “figure of 8”) on the treatment area in a homogeneous way, covering the entire surface. The movements should come out slightly from opposite sides of the perimeter of the 15 × 15 cm^2^ square. It is recommended to perform smooth and continuous movements on the treatment area for optimal results.

We suggest holding the handpiece “vertically” and maintaining contact with the skin. The only pressure on the tissue should be the pressure from the handpiece weight resting on the patient’s skin, not from the therapist. The user should slide the handpiece over the skin without applying any force, but making sure to keep the head of the handpiece in perfect contact with the skin. In fact, both handpieces are equipped with a color LED system that provides information about the handpiece–skin coupling: if the LED is green, the coupling is optimal, and the system works well; if the light turns yellow or red, there will be no adequate adhesion between the handpiece and the skin (yellow LED = less than 70% coupling; red LED = no coupling, and microwaves stop delivery).

The user’s opposite hand (not used to hold the handpiece) will be needed to perform a useful massage to “distract” the patient’s perception. Moreover, the persistent passage of the free hand on the skin, alternating with the handpiece, allows one to even out the heat, drain liquids, and provide constant feedback that is useful for monitoring the skin temperature.

We recommend starting the treatment with conservative settings (low power), and then incrementally adjusting them upwards as needed, while monitoring the patient’s tolerance, as outlined in the protocols. The objective is to elevate the subcutaneous temperature gradually and uniformly. It is preferable to treat the area with limited power, to ensure even heating over a prolonged period, rather than rapidly elevating the temperature for a short duration. Moreover, using high power could lead to less control over the temperature increase and, potentially, more side effects.

If the area to be treated is smaller than 15 × 15 cm, it is advisable to proportionally reduce the dose and/or power.

#### 5.3.2. Monitoring the Treatment

The use of Onda Coolwaves could cause a localized increase in superficial temperature and slight discomfort, which must be monitored.

When redness/slight erythema during or after treatment is observed in some areas, it is advisable to avoid further action in the same zone until the area starts to lighten again.

In case of intense erythema, sensation of heat, vesiculation, and deep pain, interruption of the treatment is advised. In this case, microwave emission should be interrupted, while continuing to massage the handpiece on the painful area, to cool it down, for 5–7 s. Treatment may be started again with a faster and wider movement, avoiding the area of discomfort for the following steps: repeat the cooling technique up to the maximum number of times recommended—three times (for a maximum of 5/6 s)—during the treatment of the same square, beyond which it is recommended to continue the treatment after reducing the power by 10 W. In the case of discomfort, it is also advisable to apply additional Vaseline oil and move the handpiece faster and wider. During treatment, the patient may experience slight discomfort, a mild tingling sensation, and temporary redness, due to the energy causing subcutaneous heating. The power delivered should be adjusted according to the patient’s pain sensitivity and the specific tissue being treated. The aim is to ensure that the treatment is effective while avoiding excessive pain. The skin may be softer to the touch. While moving the handpiece on the skin and placing their hand on the skin, the user should experience uniform heating across the treated area.

An important aid in monitoring the correct proceeding of the treatment is provided by a thermal viewer system. This is an advanced technology that guides and supports the user regarding how to manage the treatment in an enhanced and easy way. For this reason, its use is highly recommended, especially for beginners. Through the thermal camera, framing the treatment area with the handpiece, it is possible to see the real-time temperature on the skin, monitoring surface variations caused by the treatment. It provides real-time info on treatment quality and uniformity, and it represents a valid aid in assessing whether the speed with which the handpiece is moved on the area to be treated is correct or not.

Among the numerous software applications available, the “FLIR ONE” app provides some tools such as spot meters, regions of interest, and a color palette that “translates” the temperature into a specific color on the live image displayed. It is suggested to measure the surface temperature: this should never exceed 40–41 °C.

By carrying out fluid and continuous movements on the treatment area, the aim is to have a gradual and homogeneous variation in the temperature, and to observe a uniform color in correspondence with the area to be treated on the live image of the thermal camera. In addition, through the thermal viewer system, it is possible to correctly evaluate when the temperature drops in order to restart treatment, without the risk of erroneously anticipating it between interval stops.

The use of some water-based emulsions and improper use of the thermal viewer system may falsify the surface temperature measurement, despite the actual increase in subcutaneous temperature.

Additionally, consecutive movements and high power settings can rapidly escalate both pain and temperature. Similarly, if the handpiece targets excessively small areas, the resulting temperatures may be too high, potentially leading to undesirable effects.

On the other hand, if larger areas are treated (or if the areas are treated for a shorter time), the temperature may increase locally without reaching the endpoint threshold, resulting in a poorly effective treatment. Therefore, it is not recommended to extend movements beyond each designated treatment area, as doing so could render the treatment ineffective. The heating of subcutaneous tissue can vary in duration depending on the power used; however, it is also influenced by the scope of the movements.

#### 5.3.3. Possible Adverse Effects

Mild side effects, such as itching, numbness, hardness, warmth, tenderness, redness, swelling, burns, bruising, nodules, and blisters on the treatment area, are typically temporary and usually resolve within a few days following treatment. 

Improper use of the system (such as excessive handpiece pressure or treatment parameters that are too high) or incorrect patient evaluation increase the risk of adverse effects. The most common causes of the main undesirable effects that may occur are explained below to prevent them or, if necessary, intervene promptly.

If the patient complains of an excessive heat sensation, the operator should immediately interrupt the emission of microwaves and continue to apply the handpiece to the area, keeping it in contact with the skin to ensure adequate cooling.

Any soreness in the area after treatment may be due to excessive handpiece pressure by the operator, or excessive inflammation caused by an excessive parameter selection.

Hematomas may be due to excessive handpiece pressure by the operator, excessive capillary fragility of the patient, or the use of anticoagulants before treatment. Drainage of hematomas, followed by compression, is crucial, and may necessitate multiple treatments. Rapidly forming hematomas could suggest significant vascular bleeding, which would require immediate direct compression.

Headache, fatigue, exhaustion, and fever after treatment may be produced by various factors: an excessive number of areas treated in a single session; a patient with a body temperature below the average, who is therefore more affected by the increase in temperature induced by the treatment; and a state of anxiety/fear in the patient during treatment.

Cases of allergies to Vaseline with consequent dermatitis are very rare.

Adverse effects at the treatment site, such as skin and fat tissue necrosis, skin contour irregularities, and asymmetry, may arise from improper use of the system. This includes excessive energy levels, incorrect fat tissue assessment, or overly aggressive fat removal and destruction of fibrous bands. Seromas can develop when a potential space is created and fills with fluid; these typically occur within two weeks postoperatively. In case the risk of the onset of seroma formation is identified, proper garment fitting is essential [14] (recommended).

Nodules can develop just below the skin and in internal tissues. A nodule is a growth of abnormal tissue following an inflammation process, often due to an infection, an autoimmune reaction, or high power settings. These nodules are considered typical for fat treatments and have been reported to resolve, on average, within 78 days (in very rare cases, permanent nodules may form). It is likely that these nodules are derived from fat lysis, as has been observed from employing ultrasound. These are, possibly, fat necrosis aggregates [14]. Drainage massage after fat removal treatment is recommended to reduce the risk of nodule formation. In the case of nodule formation, subsequent treatment should be postponed after the nodule has disappeared. To make nodules disappear sooner, we recommend undergoing lymphatic massage two to three times per week for at least three weeks.

Patient pain feedback should be used as the basis for choosing the energies in each treatment area for every patient. This is very helpful to limit side effects, enhance patient safety, and maximize results.

### 5.4. Post-Treatment Recommendations

A lymphatic drainage massage should be performed immediately after using Onda Coolwaves, acting on the microcirculation and enabling drainage of the fluids that move during the treatment. The drainage action eliminates superficial fluid retention, thus banishing the “orange peel” appearance. The dermis is thus improved, and accumulated toxins are removed, which is immediately appreciable by changes in skin tone and color. It is advisable not to treat the same area again until 15 days have elapsed.

The patient should avoid direct sun exposure for two days after the treatment to prevent erythema ab igne.

If the skin appears slightly pink or red on the treated areas, the patient should avoid using hot water until the erythema has subsided.

A healthy diet and moderate physical activity are recommended.

Follow-up visits at one month after treatment are also advisable.

## 6. Discussion and Conclusions

Nowadays, fat adiposity and cellulite treatments represent a frequent patient request for aesthetics; often, this request is due to reduced physical activity and a lack of access to noninvasive treatments.

Invasive techniques, such as liposuction, remain the gold standard for treating localized fat deposits. However, skin laxity and cellulite may benefit from various less invasive techniques and treatments. In fact, the unwanted appearance of wavy or almost knotty skin, associated with cellulite and skin laxity, does not disappear with surgical liposuction. Treatment with microwaves has been shown to mold shapes and volumes, making them more regular, without modifying the skin surface. Various therapeutic modalities have been proposed for the treatment of cellulite, skin laxity, and adipose tissue.

Among these, cosmetics, lymphatic drainage massage, radiofrequency, ultrasound, laser therapy, carboxytherapy, intense pulsed light, subcision vibration/oscillation platform therapy, and, most recently, extracorporeal shockwave therapy (ESWT) or acoustic wave therapy (AWT), have shown efficacy in different studies [15,16,17,18,19,20,21].

Unfortunately, most treatments only demonstrate a potential for adiposity and cellulite improvement in the short term, despite a high number of treatment sessions. Therefore, demand for noninvasive and long-lasting treatments has increased, and new medical devices have been developed as a result. Among these, microwave technology is a noninvasive technique, introduced to the market with the Onda Coolwaves (DEKA, Florence, Italy) system, which is based on the delivery of microwaves into the deeper skin layers.

Microwaves are part of the RF spectrum (frequency range between 1 and 300 GHz). This spectrum has recently been used in different medical fields, including surgery, oncology, and dermatology.

The release of 2.45 GHz microwaves to the skin’s hypodermal layers demonstrated selective heating of adipocyte cells without affecting the dermal–epidermal layers. This process resulted in complete metabolic macrophage-induced adipolysis, which was clinically demonstrated by a reduction in subcutaneous adipose tissue. Additionally, cellulite was improved through the solubilization of collagen septa caused by the heat, leading to the contraction of dermal collagen fibers and an overall improvement in the external skin architecture [3,22].

Controlled heating thus stimulates the solubilization of deeper collagen fibers, activates fibroblasts, and promotes collagen remodeling.

Based on these findings, a significant improvement in cellulite, with a significant decrease in the number and depth of depressions, has been shown by Bennardo et al. in recent research [8].

To summarize, microwave technology, specifically the Onda Coolwaves system, represents a promising noninvasive alternative for treating skin laxity and cellulite, particularly for patients seeking less invasive options than traditional surgical methods like liposuction. The key findings of our review suggest that this technology can effectively target subcutaneous fat and improve skin texture with minimal disruption to the surrounding tissues. Clinically, this translates into a potential reduction in treatment times, a broader patient eligibility range, and an increase in patient satisfaction, due to its noninvasive nature and visible results. Practical applications of this technology may include integrating microwave treatments into multimodal aesthetic practices to address various concerns related to skin laxity and localized adiposity.

In conclusion, this compendium aims to propose the ideal parameters, patient selection, and treatment methodology, based on the most recent data available, in order to provide safe and effective treatment protocols.

While the preliminary results demonstrate the potential efficacy of microwave technology in reducing subcutaneous adiposity and improving skin laxity and cellulite, this study has limitations, including a lack of long-term data on safety and effectiveness. Future research should focus on conducting larger-scale, randomized controlled trials with extended follow-up periods to evaluate sustained outcomes and potential side effects. Additionally, further studies are needed to identify the optimal patient profiles and treatment parameters to maximize benefits while minimizing risks.

Future research findings may suggest changes to the conclusions or recommendations in this report.

## Figures and Tables

**Table 1:** Anamnesis and data collection.

Personal data
Previous or present illnesses
Pathologies or syndromes
Pharmacological treatment in progress
Undergone surgeries
Prostheses
Allergies
Weight and eating habits (it is suggested that BMI be calculated to assess any problems related to the patient being overweight or obese)
Previous cellulite/tightening/fat reduction treatments and any results obtained
Family medical history

**Table 2:** Visual examination should evaluate the following.

The area selected to be treated
The type and condition of the imperfection or pathology (localized adiposity, cellulitis, skin laxity)
Signs of venous insufficiency in the lower limbs
Phototype, skin hydration, any lesions, capillaries, dermatitis, or burns in the area
Any eventual body asymmetries

**Table 3:** Main and relative contraindications to the treatment.

**Main contraindications to the treatment that should be considered:**
Heart failure
Heart implants/pacemakers
Current malignancy or malignancy in the past 5 years
Severe vascular diseases (e.g., active phlebitis, thrombophlebitis, phlebothrombosis, patients with clotting disorders/hemorrhagic diathesis)
Transplant patients with a transplanted organ
Pregnancy and breast-feeding until 10 months after delivery
Infectious diseases (e.g., hepatitis B and C)
Kidney and liver failure/dysfunction
Subjects with decompensated type I and II diabetes
Recognized sensitivity to the device
Patients with deep brain stimulation implants
Patient with devices for sacral neuromodulation
**Relative contraindications to the treatment include:**
Skin pathologies (rashes, inflammation, infection, hematoma, wounds, etc.; the treatment area must be free of any open lesions)
Skin disorders stimulated by heat
Previous cancers (more than five years before)
Anticoagulant and anti-aggregant treatments (possibility of persistent erythema)
Use of anti-inflammatory drugs with steroids one week before the treatment (using these drugs in the week following treatment is also advised against)
Use of retinoids, antioxidants, and skin nutrition supplements within a month from the treatment
History of phlebitis or thrombosis
Presence of telangiectasias in the area to be treated
Presence of lipomas in the area to be treated
Subjects with autoimmune diseases
Breast-feeding (beyond 10 months after delivery)
Varicose veins
Compensated diabetes
Predisposition for the formation of keloids or abnormal healing
Hypertriglyceridemia and hypercholesterolemia
Alteration in blood pressure
Presence of fibrotic tissue and particularly lax tissue
Since ongoing feedback is required during the procedure, if the patient has nerve insensitivity to heat anywhere in the treatment area, microwave treatment is not advised
Previous treatments with fillers or botulinum toxin in the area to be treated with microwaves could reduce the effect of the injectables. To avoid this possibility, patients can postpone treatment until the previous aesthetic treatment has finished its effect (for botulinum toxin, about 6 months from injection; for fillers, depending on their absorption characteristics, from about 3 months for ultra-fast-absorbing fillers, and up to about 12 months or more for slow-absorbing fillers)
Patients with a BMI > 30
Microwave treatment is not recommended for obese patients. For this reason, a BMI > 30 is generally considered an exclusion criterion for the treatment. However, this criterion is only indicative and does not exclude the need to carry out a careful assessment of each specific case. More than the total amount of fat mass, it is important to consider its distribution. In fact, it is possible to have patients with a higher BMI but equally treatable areas or, on the contrary, patients with a BMI < 30 but who are not treatable. Let us examine two practical examples: (1) a patient with an androgynous morphotype, BMI = 32, and obesity concentrated in the abdominal and upper body area, but “normal” lower limbs, can be treated on the thighs or buttocks in the case of cellulite; (2) in a patient with a gynoid morphotype, BMI = 29, and very big legs with a skinny upper body, microwave treatment of the lower limbs is not possible
Underage patients
Patients with unrealistic expectations

**Table 4:** Areas to be excluded from the treatment.

Head and neck, using the standard handpieces (deep and shallow). The only pre-platysma fat that can be treated is in the submental area, starting from 1.5 cm below the lower border of the mandible up to the hyoid bone. This is to avoid treating the area of the marginal mandibular nerve, arteries and veins
Genitalia
Cardiac, décolleté, and breast areas
Back (except for the bra line)
Leg (under the knee), foot, forearm, and hand
Belly button: due to its characteristic conformation, the umbilical area does not allow adequate skin cooling using the surface of the handpieces. For this reason, the user must be very careful and avoid going over this area. Otherwise, the lack of proper cooling could lead to the accumulation of hot liquids, with a consequent risk of burns
Bone protrusions
Mucous membrane
Lymph node stations
Tissue with a limited thickness of the subdermal fat layer (<1 cm). This is recommended in the case of localized fat treatment. Make sure to identify areas with a skinfold thicker than 2 cm (the subdermal fat layer must be at least 1 cm thick) and thinner than 5.5 cm (the thickness should be assessed with the aid of instrumental examinations, like a plicometer measure). In the case of patients with skin laxity, it is possible to act even when the thickness of the subdermal fat layer is <1 cm. For example, a very thin patient with postpartum skin laxity can still be treated with Onda by using the small handpiece and lowering the parameters (maximum dose 130,000 J per quadrant and maximum power 130–140 W)
Areas with acute inflammatory processes (such as rashes, inflammation, infection, hematoma, wounds, etc.)
Open wounds
Areas with permanent implants, such as metal/plastic plates, prosthesis, and screws, or injected chemical or autologous substances or fat injections
Femoral, subclavian, and brachial arteries and veins
Varicose veins
Pharmacologically anesthetized areas (absence of patient feedback on pain)
Fractures (even if in the process of healing)
Areas with sensibility reduced or absent
Ischemic tissues of individuals with vascular disorders whose blood circulation is insufficient to cover increased metabolic requirements (risk of necrosis)
Spine areas subjected to laminectomy
Hernias
Lipomas
Fibrotic tissue and particularly lax tissue
Subjects with any body piercing in the treatment area

**Table 5:** Features of the handpieces.

Features of the Shallow Handpiece	Features of the Deep Handpiece
0.7 cm deep focalization of the heating	1.2 cm deep focalization of the heating
Integrated cooling (up to 5 °C)	Integrated cooling (up to 5 °C)
Recommended for skin tightening, superficial cellulite, and first 1 or 2 sessions in case of fibrotic cellulite, thin layer of fat.	Recommended for localized fat and deep cellulite

**Table 6:** Handpiece selection according to patient condition.

• Patient’s localized adiposity as a dominant problem: deep handpiece.
• Patient with cellulite as a dominant problem (understood as cases where cellulite is in the early stages and is of a predominantly edematous type): shallow handpiece.
• Patient with laxity as a dominant problem (such as the abdomen after pregnancy): shallow handpiece.
• Patient with mixed localized adiposity and cellulite (fibrotic): the first or even second sessions are performed with the shallow handpiece, and then continued with the deep one.
• The treatment of an advanced fibrotic cellulite can be more difficult if, in the first sessions, the deep handpiece (which would act on the fat cells of the hypodermis) is immediately used. In practice, using the deep handpiece in the presence of considerable subcutaneous fibrosis acts as a shielding action, and it is likely that the light on top of the handpiece turns either yellow or red due to a nonoptimal coupling. This is the reason why it is more efficient to spend the first sessions using the shallow handpiece, which, by lowering its depth, finds less resistance. Coolwaves can, thus, effectively act on the fibrotic septa at their anchor points close to the dermis (note: septa are usually less fibrous at their endpoint, close to their anchor point, than in the central part, which is much harder and less elastic). The heat produced spreads towards the lower part of the septa, thus triggering the solubilization process that has been histologically observed [6]. Once the septa have been “softened”, it is possible to use the deep handpiece, which will be able to penetrate more easily in depth, no longer encountering strong resistance from the fibrous branches.
• Mixed cellulite and laxity: shallow handpiece.
• Patient with mixed localized adiposity and laxity: the first sessions are carried out with a deep handpiece, followed by the shallow handpiece. The initial action of the deep handpiece acts on fat, but it also has a secondary action on laxity. When the volumes have been reduced, it will be possible to focus more on laxity, with a more superficial targeted action.
• Patient with localized adiposity, cellulite, and laxity: the patient should be well assessed, considering which components are most important, to decide whether to start with the deep handpiece or with the shallow, according to the rationale explained above.

## Data Availability

The original contributions presented in this study are included in the article. Further inquiries can be directed to the corresponding author.
